# A 5′ UTR Mutation Contributes to Down-Regulation of *Bbs7* in the Berlin Fat Mouse

**DOI:** 10.3390/ijms232113018

**Published:** 2022-10-27

**Authors:** Kourosh Mohebian, Deike Hesse, Danny Arends, Gudrun A. Brockmann

**Affiliations:** Albrecht Daniel Thaer-Institut für Agrar- und Gartenbauwissenschaften, Humboldt-Universität zu Berlin, Unter den Linden 6, 10099 Berlin, Germany

**Keywords:** dual luciferase, *jObes1*, gene regulation, transcription, SNP, obesity

## Abstract

The Bardet–Biedl Syndrome 7 (*Bbs7*) gene was identified as the most likely candidate gene causing juvenile obesity in the Berlin Fat Mouse Inbred (BFMI) line. *Bbs7* expression is significantly lower in the brain, adipose tissue, and liver of BFMI mice compared to lean C57BL/6NCrl (B6N) mice. A DNA sequence comparison between BFMI and B6N revealed 16 sequence variants in the *Bbs7* promoter region. Here, we tested if these mutations contribute to the observed differential expression of *Bbs7*. In a cell-based dual-luciferase assay, we compared the effects of the BFMI and the B6N haplotypes of different regions of the *Bbs7* promotor on the reporter gene expression. A single-nucleotide polymorphism (SNP) was identified causing a significant reduction in the reporter gene expression. This SNP (rs29947545) is located in the 5′ UTR of *Bbs7* at Chr3:36.613.350. The SNP is not unique to BFMI mice but also occurs in several other mouse strains, where the BFMI allele is not associated with lower *Bbs7* transcript amounts. Thus, we suggest a compensatory mutation in the other mouse strains that keeps *Bbs7* expression at the normal level. This compensatory mechanism is missing in BFMI mice and the cell lines tested.

## 1. Introduction

More than one-third of the world’s adult population is overweight, and the incidence is further increasing [1]. In recent years, in particular, childhood obesity has become a global epidemic, occurring at high rates all over the world [2]. In addition to malnutrition, heritability plays a major role in the development of obesity. The contribution of genetic predisposition to the onset of obesity is particularly high in children [3,4]. The genetics of metabolic traits such as obesity are complex and multi-faceted; therefore, finding a single causal locus cannot always be expected when dealing with such complex traits. However, the identification and understanding of each contributing factor will help to unravel the complex pattern and thereby assist in understanding the development of obesity.

The Berlin Fat Mouse Inbred line (BFMI) carries a natural mutation causing juvenile obesity. Males and females of the BFMI line have a high body fat content already under a standard diet at the young age of 6 to 10 weeks [5,6]. In addition, BFMI mice show several features of the metabolic syndrome such as impaired lipid metabolism and reduced insulin sensing [7,8,9,10]. Therefore, BFMI mice are a suitable model to investigate genetic determinants and mechanisms contributing to juvenile obesity.

Previous research in a F2 crossbred population between obese BFMI and lean C57BL/6NCrl (B6N) mice identified the *jObes1* locus on chromosome 3 as the main driver for obesity, which explains approximately 40% of the variation of the ratio between total body fat mass to total body lean mass [11]. This locus acts recessively; only mice homozygous for the mutant allele become obese. Subsequent fine mapping of the *jObes1* locus in an advanced intercross line and complementation tests with knockout mouse lines identified the Bardet–Biedl Syndrome 7 (*Bbs7)* gene as the most likely causal gene for the juvenile obesity phenotype of BFMI mice [12].

The Bardet–Biedl Syndrome is a ciliary disorder that occurs in the human population at an incidence of 1 out of 100,000. There is a significant interest in finding and characterizing the different *Bbs* genes. Among the *Bbs* genes, *Bbs5*, *Bbs6*, *Bbs7*, *Bbs8*, *Bbs9,* and *Bbs11* are expressed in adipose tissue [13] and *Bbs2, Bbs5, Bbs7,* and *Bbs9* expression was reported in the brain [14]. Mutations in these genes might have a direct impact on adipogenesis and adipose tissue functions, leading to the overweight phenotype in Bardet–Biedl Syndrome patients [13].

The BBS7 protein is among the key proteins for the formation of the BBSome [15]. Genetic defects in the *Bbs7* gene contribute to Bardet–Biedl Syndrome in humans [16] and mice [17]. The Bardet–Biedl Syndrome 7 protein (BBS7) interacts with several other proteins to form the BBSome, an intracellular transport complex that is essential for the biogenesis of ciliary membranes and the transport of proteins to the cell membrane [18]. Defects in any of the BBS proteins impair the formation and function of the BBSome [17]. Knockdown studies of BBSome subunits showed the same direction of effects, namely, the loss or impairment of the formation of the BBSome [15,19]. For instance, studies on *Bbs1*^−/−^, *Bbs2*^−/−^, and *Bbs4*^−/−^ knockout mice provided evidence for the significant role of the BBSome for the translocation of G protein-coupled receptors to the cell membrane of primary cilia in neurons [20]. This was shown for the leptin receptor [21], the somatostatin receptor 3 [22], the dopamine receptor [23], and the melanin-concentrating hormone receptor 1 [20]. The knockdown of *Bbs7* in mice led to reduced axonal growth and brain abnormalities [24]. The missing BBS7 protein affected motile cilia structures in the ependymal layer of the brain [17]. Homozygous *Bbs7* knockout mice at 12 weeks of age were significantly heavier than control males and females due to increased food intake [17].

The Bardet–Biedl Syndrome in humans is a recessive disorder [25]. This is also the recessive mode of inheritance in mice. Therefore, homozygous and heterozygous *Bbs7* knockout mice do not show the obese phenotype of wild-type mice. Unlike in the *Bbs7* knockout mouse, where the gene is not expressed, *Bbs7* is expressed in BFMI mice throughout their development, but the expression is reduced due to a genetic defect at the *Bbs7* locus in BFMI mice. The mechanism of reduced expression leads to a milder obese phenotype in BFMI mice than in *Bbs7* knockout mice. Finding the causal mutation for the low expression of *Bbs7* in BFMI would improve our understanding of the contribution of defects in gene regulation to the pathogenesis of diseases. In humans, many BBS protein variants have been associated with the Bardet–Biedl Syndrome [26]. Nonetheless, there are cases of Bardet–Biedl Syndrome which cannot be explained by protein variants. Therefore, the impairment of gene regulation could be amechanism in addition to the loss of protein function which might cause obesity in patients with Bardet–Biedl Syndrome. Yet, the consequences of DNA sequence variants in regulatory regions of *Bbs* genes have not been examined in humans so far.

In our BFMI mouse model, we could exclude the BBS7 protein variant as a causal factor for obesity, since this protein variant occurs in several mouse strains which are not obese (Ensembl release 102) [27]. In contrast, reduced gene expression of *Bbs7* in different tissues of BFMI mice in comparison to lean B6N control mice provided a hint on down-regulation as a potential mechanism causing obesity in BFMI mice [12]. The comparison of the DNA sequences in the promoter region of *Bbs7* (36.613.319–36.614.267 Ensembl release 102) between BFMI and B6N identified 16 DNA sequence variants [27], which could potentially influence the transcription.

The current study aimed to further elucidate the molecular mechanism that causes juvenile obesity in BFMI mice. Here, we tested DNA sequence variants in the promoter region of *Bbs7* that occur between BFMI and B6N mice in reporter gene assays in order to identify the causal mutation underlying the expression differences of *Bbs7* seen between the two mouse strains.

## 2. Results

### 2.1. Effects of Variants in the Bbs7 Promoter Region on the Expression of the Luciferase Gene

**Figure 1:**
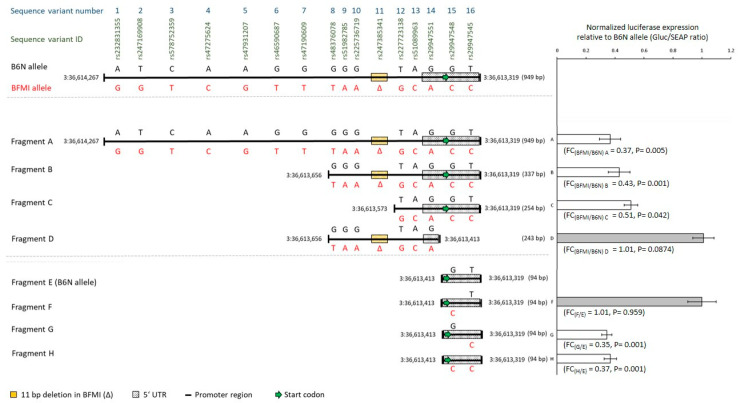
On the left side, promoter fragments are shown that were tested for their effect on the expression of luciferase as the reporter gene in the dual-luciferase plasmid (ZX-103). The full-length fragment A comprises the whole Bardet–Biedl Syndrome 7 (*Bbs7*) promoter region of 949 bp between 3:36,613,319 and 3:36,614,267. The 5′ UTR is shown with a patterned black and white box. Fragments B, C, and D are shorter cloned promoter fragments. Fragments E through H (3:36,613,319 to 3:36,613,413) were synthesized. E comprises the shortened C57BL/6NCrl (B6N) haplotype; F, G, and H contain Berlin Fat Mouse Inbred (BFMI) alleles in different combinations. On top, all 16 sequence variants across the promoter fragment are shown with their ID and numbers from 1 to 16. Above every fragment, the B6N alleles are depicted (black), below the BFMI alleles (red). On the right side, a bar chart shows the normalized luciferase expression (GLuc/SEAP ratios) of the tested BFMI promoter variants versus B6N as the reference. Significantly reduced expression was evident for BFMI fragments A, B, and C (FC_(BFMI/B6N)A_ = 0.37, FC_(BFMI/B6N)B_ = 0.43, FC_(BFMI/B6N)C_ = 0.51), but not for D. Testing the short synthesized fragments provided evidence for the importance of single-nucleotide polymorphism (SNP) 16 (rs29947545). The BFMI allele of SNP rs29947545 is required (fragments G and H) for significantly reduced expression (FC_(G/E)_ = 0.35, FC_(H/E)_ = 0.37, FC_(F/E)_ = 1.01). Normalized luciferase expression (GLuc/SEAP ratio) was plotted as mean ± standard deviation of triplicates.

The tested promoter region of 949 base pairs (3:36,613,319 and 3:36,614,267 Mb) comprises 856 base pairs upstream of the *Bbs7* start codon and 159 base pairs of the 5′ UTR (66 base pairs of the 5′ UTR in the upstream sequence of the start codon). The region contains 16 sequence variants between BFMI and B6N; 15 single-nucleotide polymorphisms (SNPs) (12 upstream of the 5′ UTR in the promoter region, 3 in the 5′ UTR), and an 11 bp deletion upstream of the start codon (Figure 1). In the following, we use the numbers 1 to 16 for the sequence variants to facilitate the understanding of their effects on reporter gene expression.

First, the effect of BFMI and B6N haplotypes of the full-length promoter region with all 16 sequence variants (fragment A, Figure 1) on the reporter gene expression was tested. The normalized expression of the luciferase gene behind the BFMI promoter was reduced to a fold change of 0.37 compared to the B6N promoter (fragment A, FC_(BFMI/B6N)A_ = 0.37, Figure 1). The magnitude of the reduction in the reporter gene expression with the BFMI promoter is consistent with the reduction in the expression of *Bbs7* in 10-week-old BFMI versus B6N mice in the whole-brain (FC_(BFMI/B6N)_ = 0.39) [12].

The BFMI promoter variants of the two shorter fragments B and C, containing the sequence variants 8 to 16 and 12 to 16, respectively, showed also a reduction in the expression of the reporter gene. The reduction was in the same range as for the full-length BFMI fragment A (FC_(BFMI/B6N)B_ = 0.43, FC_(BFMI/B6N)C_ = 0.51, Figure 1). The sequence variants 1 to 11 in the region between 3:36,613,573 and 3:36,614,267 Mb, which occur in fragments A and B only, could be excluded from being causal for the difference in expression. This is due to the fact that the full-length promoter as well as the shorter BFMI variants of fragments A, B, and C cause a reduction in the reporter gene expression. The excluded region also comprises the 11 bp deletion upstream of the 5′ UTR of *Bbs7* in BFMI mice (Figure 1) [27]. After testing the fragments A, B, and C, sequence variants 12 to 16 remained to be tested for gene expression.

The short promoter fragment D contains the sequence variants 8 to 14, but not 15 and 16. This fragment was designed to test whether the two sequence variants 15 and 16 which are located in the 5′ UTR downstream of the 11 bp deletion are necessary for the reduction in expression in BFMI (Figure 1). The reporter gene assay did not show any difference in the expression between the BFMI and B6N promoter fragments D (FC_(BFMI/B6N)D_ ≈ 1). Based on this result we dismissed the three sequence variants 12, 13, and 14 between 3:36,613,413 and 3:36,613,573 Mb from being causal and focused on the remaining two sequence variants 15 and 16 which are located in the 5′ UTR.

To figure out which of the two remaining sequence variants 15 and 16 are causal for gene expression differences, the four fragments E to H were synthesized as oligonucleotides which all comprise the sequence between 3:36,613,319 and 3:36,613,413 Mb. Fragment E contains the reference sequence of B6N, fragment F the BFMI allele of sequence variant 15, fragment G the BFMI allele of sequence variant 16, and fragment H the BFMI alleles of sequence variants 15 and 16. Normalized expression levels of the luciferase reporter gene showed consistently a reduction in expression when the BFMI allele C of sequence variant 16 was present in the sequence. This was the case for the BFMI fragments G and H compared to the B6N fragment E (FC_(G/E)_ = 0.35, *p* = 0.001; FC_(H/E)_ = 0.37, *p* = 0.001, Figure 1). Moreover, no difference was evident in the normalized expression between the fragments F and E (FC_(F/E)_ = 1.01, *p* = 0.959, Figure 1). Therefore, sequence variant 15 was excluded from being causal.

The exclusion of all sequence variants except number 16 led to the conclusion that SNP rs29947545 with the allele C in BFMI is the only candidate that can cause the reduction in expression in the reporter gene assay.

The effects reported above were observed in HEK 293A cells, which originate from human embryonic kidney, which are easy to transfect, and which express *Bbs7*. To verify those results in a murine cell system, 3T3-L1 cells, which originate from murine preadipocytes, were used. However, the transfection efficiency in the 3T3-L1 cells was very low compared to HEK 293A cells. Since fewer cells were transfected, lower luciferase expression levels were observed. Nonetheless, the normalized luciferase expression of fragments E, F, G, and H in 3T3-L1 cells showed similar expression levels of luciferase in 3T3-L1 cells when compared to HEK 293A cells with reduced expression in fragments G and H. These observations further underline the findings in HEK 293A cells (Supplementary File S1, Figure S1).

### 2.2. Occurrence of the Identified BFMI SNP Allele in Other Mouse Inbred Strains

To examine the potential function of the identified SNP rs29947545 for obesity, we checked in which other inbred mouse strains the BFMI allele (C instead of T) occurs. Surprisingly, 30 out of 43 (69%) commonly used inbred strains for which whole-genome sequence data are available [28] carry the BFMI allele (C) (Supplementary File S3). Frequently, they do not only carry the C allele at rs29947545, but the whole BFMI haplotype of neighboring SNPs. Yet, most of those strains are lean. Therefore, these findings point toward a more complex mechanism in mice which could include an additional genomic factor interacting with the SNP.

To further clarify a potential interaction of the SNP rs29947545 with another genomic factor, we investigated the strains AKR (prone to diet-induced obesity (DIO)) and SJL (resistant to DIO). These two strains have a *Bbs7* promoter similar to BFMI (Table 1) but have not been reported to develop juvenile obesity under a normal diet.

### 2.3. Bbs7 Expression in B6N, BFMI, AKR, and SJL

**Figure 2:**
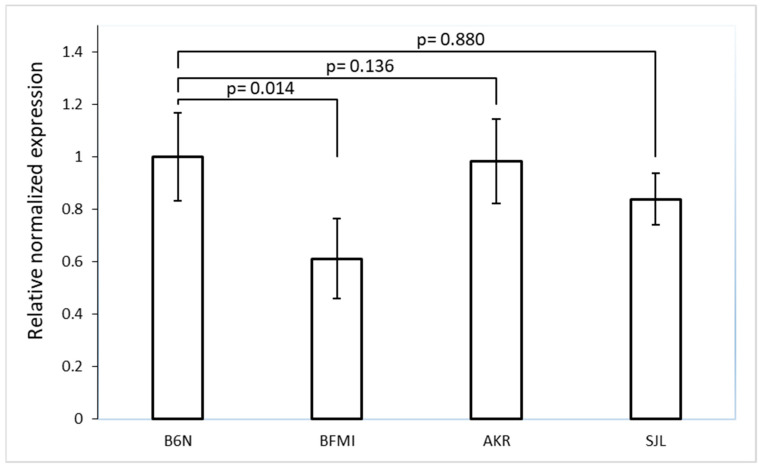
Normalized *Bbs7* expression (RT-PCR) in the brain of 6-week-old BFMI, B6N, AKR, and SJL males. Beta-actin was used as the housekeeper gene. The expression of *Bbs7* was decreased by almost 40% in BFMI versus B6N mice only (FC_(BFMI/B6N)_ = 0.61). No significant difference was observed when comparing AKR and SJL with B6N.

AKR and SJL share the *Bbs7* promoter haplotype with BFMI between 3:36,613,319 and 3:36,614,267 Mb except for variants 3 and 11 where they carry the B6N alleles. The measurement of *Bbs7* transcript amounts in tissue from the brain confirmed the previous finding of lower expression in BFMI compared to B6N [12] (fold change FC_(BFMI/B6N)_ = 0.61, *p* = 0.014, Figure 2). However, no significantly reduced *Bbs7* expression was observed for AKR (*p* = 0.880) and SJL (*p* = 0.136) compared to B6N underlining the assumption that the promoter haplotype alone is not sufficient to produce the BFMI phenotype and that additional factors are needed.

## 3. Discussion

In this study, we examined the regulatory effects of promoter variants of the *Bbs7* gene on its gene expression. The *Bbs7* promoter variants of BFMI and B6N mice were tested using dual-luciferase reporter gene assays performed in HEK 293A cells. The experiments provided clear evidence that SNP rs29947545 caused reduced expression in our luciferase assay.

Although our reporter gene assay was performed in HEK 293A cells, we ensured that the results observed in this human cell line can be replicated in a mouse cell line (3T3-L1). The 3T3-L1 cell line was chosen to reproduce our results in a murine cell line since it can be used to study the basic cellular mechanisms associated with obesity and related disorders [29,30,31]. Although transfection rates and subsequent luciferase expressions were much lower in 3T3-L1 cells, the BFMI/B6N ratios of luciferase expression observed in 3T3-L1 cells were similar to the reduction in HEK 293A cells. Therefore, we are confident that the BFMI allele C of SNP rs29947545 is causal for the reduction in expression in the transfected cells.

The SNP rs29947545 is located in the 5′ UTR of *Bbs7*, a region that is crucial for post-transcriptional regulatory mechanisms [32,33,34,35]. UTRs generally are recognized to have critical roles in post-transcriptional regulation of gene expressions, such as modulation of the transport of mRNAs out of the nucleus [28], mRNA stability [36], and subcellular localization [37]. DNA variations in the 5′ UTR, such as SNP rs29947545, could regulate gene expression through one of the mentioned post-transcriptional pathways.

Another way in which this SNP in the 5′ UTR could affect transcription levels is through disruption of an existing transcription factor binding site (TFBS) [38]. Whether a SNP potentially disrupts the binding of transcription factors (TF) or leads to a new TFBS can be valuable information. This information can help in interpreting distinct results from different mouse strains sharing the same SNP. Notably, it can help us explain the mechanisms at the molecular level by which the DNA variant directly affects it. For this reason, we used SwissRegulon [39] and PROMO [40,41] to predict if SNP rs29947545 is located at currently known TFBSs. Unfortunately, the results from the two mentioned databases were inconsistent over time. Currently, no TFBS potentially binding to the target region is given in ENSEMBL [27].

The BFMI allele C of SNP rs29947545 occurs in 69% of inbred mouse strains sequenced by the Mouse Genome Project [28]. This SNP might be causal in BFMI for the obese phenotype, but we cannot make the same assumption for the other mouse strains with the same SNP allele in the Mouse Genome Project database. Since most of the other mouse strains that also harbor the BFMI allele of SNP rs29947545 are not obese, we can state that the mutation alone is not sufficient for causing obesity.

With respect to the mechanism causing low *Bbs7* expression in BFMI mice, we identified in a cell-based system, a single SNP in the promoter region that significantly contributes to the regulation of transcription, but which is not sufficient to reduce the transcript amount in other mouse strains than BFMI. We assume that a second factor interacting with the region of SNP rs29947545 is necessary to affect the expression of *Bbs7.* This secondary factor is most likely only present in the BFMI mouse or a subset of mouse strains. The other mouse strains likely have a genetic background that does not express this secondary factor, or that contains a second genomic region that physically interacts with the promoter region where the SNP resides. This hypothesis is supported by the observation that the AKR and SJL strains which share the same SNP allele and almost the entire *Bbs7* haplotype with BFMI, did not show the reduced *Bbs7* expression seen in BFMI mice.

With the reporter assays, we could show that the specific SNP reduces expression in the tested cell lines (HEK 293A and 3T3-L1) that, therefore, behave as the BFMI mice rather than as the other mice. Hence, it could be that the other mouse strains with the same SNP (AKR and SJL) carry compensatory mutations that keep expression at the normal level, rather than additional mutations in the BFMI mice that confer the phenotype. The significant point is that mice that do not develop obesity also do not show reduced *Bbs7* expression.

Mutations in non-coding regulatory elements such as promoters and enhancers have been shown to play an important part in the evolution of gene expression [42,43]. *Cis* and *trans* effects of mutations on gene regulation are among the two main mechanisms giving rise to evolution. Any gene can be affected by *cis*, *trans*, or both effects [44]. With respect to B6N and BFMI, we provided evidence for a genetic effect controlling *Bbs7* expression. Since these mice were kept in the same controlled environment, the observed total genetic effect is the sum of all modes of genetic action, *cis* and *trans* effects. The cell-based reporter assays performed in this study capture the *cis* effects of the direct neighborhood of *Bbs7* on its expression. In contrast, *Bbs7* expression differences in tissues between BFMI and B6N represent the total genetic effect, including *trans*-acting factors. The observation that *Bbs7* expression was not significantly reduced in other mouse strains carrying the same promoter mutation as BFMI (e.g., AKR and SJL) could indicate an additional *cis* or a *trans* effect, which cancels out the *cis* effect of the identified SNP in the regulatory region of the *Bbs7* gene.

## 4. Materials and Methods

### 4.1. Mouse Populations

The Berlin Fat Mouse Inbred line BFMI860-12 (BFMI, Humboldt-Universität zu Berlin, Berlin, Germany) and C57BL/6NCrl (B6N, Charles River Laboratories, Sulzfeld, Germany) were used as DNA sources. The BFMI and B6N lines have previously been used for mapping and fine-mapping the juvenile obesity locus *jObes1* [11,12]. For real-time gene expression measurements, brains from 6 weeks-old BFMI and B6N mice were collected (4 samples each). For comparison reasons, we also used AKR/J (AKR, Technical University of Munich, Munich, Germany) and SJL/NDife (SJL, German Institute of Human Nutrition Potsdam-Rehbruecke (DIfE), Potsdam, Germany) mice (5 samples each). AKR and SJL share a similar promoter haplotype with BFMI mice. Nevertheless, AKR mice are prone to diet-induced obesity (DIO) while SJL mice are resistant to DIO.

### 4.2. Husbandry Conditions

All experimental treatments of animals were authorized by the German Animal Welfare Authorities (approval no. G0174/19). All methods were performed in accordance with the relevant guidelines and regulations of German Animal Welfare laws and also in accordance with Animal Research: Reporting of In Vivo Experiments (ARRIVE) guidelines. All mice were maintained under conventional conditions and a 12:12 h light: dark cycle at a temperature of 22 ± 2 °C. Animals had *ad libitum* access to food (rodent standard diet (V1534-000 ssniff R/M-H; ssniff Spezialdiäten GmbH, Soest, Germany)) and water.

### 4.3. DNA Extraction and Sequencing

DNA was isolated from BFMI and B6N animals for amplification and subsequent cloning following the DNA extraction protocol for mouse tail clips by Wang and Storm et al. [45]. The Illumina HiSeq platform (Illumina Inc., San Diego, CA, USA) was used for paired-end sequencing of the BFMI and B6N genomes isolated from the spleen. Reads were trimmed with Trimmomatic [46] after which reads were aligned to the mouse genome (MM10, GRCm38.p6) using the Burrows–Wheeler Aligner (BWA) [47]. The subsequent SAM files were converted to BAM files, indexed, and sorted utilizing Samtools [48,49]. Duplicate reads were removed by Picard tools v2.19.0 [50]. Subsequently, indel realignment and base recalibration was done using the GATK v4.1.0.0 [51], based on GATK best practices [52]. BCFtools [49] was used to call sequence variants.

### 4.4. Primer Design

The DNA sequence of the promoter region of *Bbs7* (chr3: 36.613.319–36.614.267, Ensembl release 102) from BFMI and B6N mice was loaded into SnapGene v5.0.7 (http://www.snapgene.com, GSL Biotech LLC, Chicago, IL, USA) to define the exact regions to be inserted into the linearized ZX-103 plasmid (GeneCopoeia™, Rockville, MD, USA) (see Figure 1). Primers were designed to contain overhangs complementary to the sticky ends of the respective restriction site of the linearized plasmid. All primer sequences are available in Supplementary File S1, Table S1 and their purpose of the use is mentioned in Table S2.

### 4.5. Generation of a Set of Promoter Fragments of BFMI and B6N

This study investigated 16 sequence variants in a 949 base pair region of the *Bbs7* promoter between 3:36,613,319 and 3:36,614,267 Mb. This region contains 159 base pairs of the 5′ UTR of *Bbs7* [27]. Fragments of the target region for subsequent cloning into the ZX-103 plasmid were generated for BFMI and B6N genotypes either by PCR (fragments A to D) or by synthesis of oligonucleotides (E to H) (Figure 1). This means that fragments A to H have always two variants, either a B6N variant inserted in a ZX-103 plasmid that carries the reference sequence or a BFMI variant of the same fragment that carries the mutations in the BFMI sequence inserted in another ZX-103 plasmid. The fragments A to H differ in their length. Depending on the length of the PCR products, the BFMI and B6N fragments were haplotypes containing all BFMI or all B6N alleles of up to 16 sequence variants within the examined region. The sequence variants were assigned to numbers 1 to 16 (Figure 1). For example, fragment A is the full-length sequence (positions 1–949), where the reporter plasmid contains a fragment with the B6N sequence from position 1 to position 949. This fragment served as the control for the complement BFMI version of fragment A. Then, there is a second complement reporter plasmid A that has the BFMI sequence for the same range (positions 1–949). Fragment B is a shorter sequence being inserted in the reporter plasmid that only contains positions 613 to 949 of the B6N sequence in one reporter plasmid and the BFMI sequence of the same position in another reporter plasmid. The same rule applies to all the reporter plasmids and their inserted fragments.

For each PCR reaction, 25 µL of DreamTaq PCR Master Mix (2X) (Thermo Fisher Scientific, Waltham, MA, USA) was mixed with 2.5 µL forward and 2.5 µL reverse primer (10 µM), 5 µL DNA (50 ng to 1 µg) and 15 µL nuclease-free water. Thermal cycler conditions were: 95 °C for 1 min for the initial denaturation followed by 40 cycles of amplification with denaturation at 95 °C for 30 s, annealing at 60 °C to 65 °C depending on the primers’ melting temperature for 30 s, extension at 72 °C for 1 min and a final extension at 72 °C for 7 min. The primer sequences and their melting temperatures are given in Supplementary File S1, Table S1.

DNA sequences were synthesized to obtain short fragments mimicking either the natural B6N sequence or the alternative BFMI sequence. These are fragments E to G (Figure 1). Synthesis was carried out in service (Eurofins Genomics Germany GmbH, Ebersberg, Germany).

### 4.6. Cloning

For cloning, we used the NEBuilder HiFi DNA Assembly Cloning Kit (New England Biolabs, Ipswich, MA, USA) comprising NEB 5-alpha Competent *E. coli* together with the plasmid ZX-103. The ZX-103 plasmid was linearized using the restriction enzymes HindIII and EcoRI (New England Biolabs, Ipswich, MA, USA). For restriction digest, we added 1 µg of the plasmid ZX-103 to a 50 µL reaction mixture consisting of 1 µL of HindIII (10 units), 1 µL of EcoRI (10 units), 5 µL CutSmart buffer, and nuclease-free water. The restriction digest reaction was incubated at 37 °C for 1 h. All inserts were designed to carry sticky ends. The assembly protocol from the NEBuilder HiFi DNA Assembly Cloning Kit was carried out to insert the target promoter sequences into the linearized ZX-103 plasmid using the recommended DNA ratio of the plasmid to insert by 1:2, with the total amount of fragment in the range of 0.03 to 0.2 pmol. After ligation at 50 °C for 15 min, the transformation was performed according to the kit’s protocol by adding 2 µL of the ligation reaction to 50 µL of NEB 5-alpha competent *E. coli* cells. The tubes containing this mixture were left on ice for 30 min before they were incubated in a 42 °C water bath for 45 s and directly put back on the ice for 2–3 min. Afterwards, 1 mL lysogeny broth (LB) culture medium was added to each mixture and incubated at 37 °C on a Thermo-Shaker with 800 RPM for 1 h. Subsequently, 100 μL of the culture was spread on LB Agar Kanamycin (50 µg/mL) plates. Kanamycin was used since the ZX-103 plasmid contains a Kanamycin resistance cassette. Plates were incubated at 37 °C overnight. The following day single colonies were picked for further use.

### 4.7. Plasmid DNA Isolation

Plasmid DNA was isolated from bacteria culture and purified using the GeneJET Plasmid Midiprep or Miniprep Kit (Thermo Fisher Scientific, Waltham, MA, USA). Both kits use silica-based membrane filters with spin columns. The concentration of the purified plasmid DNA was measured on a NanoDrop™ One/One^C^ device (Thermo Fisher Scientific, Waltham, MA, USA). For the subsequent reporter gene assay, the concentration of recombinant plasmids was adjusted to 500 ng/µL.

### 4.8. Cell Culture

For the reporter gene assay, we used HEK 293A cells. They were cultured in high-glucose, HEPES-supplemented Dulbecco’s modified Eagle medium (DMEM) (Thermo Fisher Scientific, Waltham, MA, USA), with 10% (*v*/*v*) fetal bovine serum (FBS) (Thermo Fisher Scientific, Waltham, MA, USA) and 0.2% Penicillin-Streptomycin (Sigma-Aldrich, St. Louis, MO, USA). 10^5^ cells per well of a standard 24-well plate were seeded in 0.5 mL medium. Prior to transfection, cells were incubated at 37 °C in 5% (*v*/*v*) CO_2_-enriched air for 24 h. The medium was changed after the 24 h incubation before the cells were transfected.

The 3T3-L1 cell line was also used with the same conditions and treatments as the HEK 293A cells. They were seeded in standard 24-well plates with 10⁵ cells per well in 0.5 mL medium. As expected, the transfection efficiency for the 3T3-L1 cell line was low given that it is an embryonic fibroblast cell line. We performed the dual-luciferase experiment on the transfected cells using the plasmids containing E to G fragments (Supplementary File S1, Figure S1, and Supplementary File S2).

### 4.9. Transfection

Transfection of HEK 293A and 3T3-L1 cells with 500 ng plasmid DNA was done with the transfection reagent EndoFectin™ Max (GeneCopoeia™, Rockville, MD, USA) according to the manufacturer’s protocol. For the dilution gradient of plasmid DNA, Opti-MEM™ I Reduced Serum Medium (Thermo Fisher Scientific, Waltham, MA, USA) was used. 2 µL transfection reagent was used for each well. Transfected cells were incubated for 48 h; the medium was not changed after transfection.

### 4.10. Dual-Luciferase Assay

The dual-luciferase reporter system Secrete-Pair™ Dual-Luminescence Assay Kit (GeneCopoeia™, Rockville, MA, USA) was used to investigate the effect of the inserted promotor fragments on the activities of Gaussia Luciferase (GLuc) and Secreted Alkaline Phosphatase (SEAP). The GLuc reporter gene is located downstream of the multiple cloning site of the ZX-103 plasmid where the promoter fragments were inserted. The experimental control gene SEAP is located downstream of a Cytomegalovirus (CMV) promoter approximately 600 bp downstream of the GLuc gene. Luciferase and SEAP activity was measured 48 h post-transfection using the GL-H and AP buffer, respectively.

Signal normalization was done by calculating the ratio of GLuc/SEAP activity, in order to eliminate the effects of transfection efficiency differences (Supplementary File S2). Fold change (FC) was calculated to explain how much the luciferase expression changed between the BFMI and B6N promoter variants. Tecan’s Infinite 200 plate reader (Tecan, Männedorf, Switzerland) was used to measure the luciferase activity (reporter gene) and SEAP (experimental control). All the luciferase experiments in transfected HEK 293A cells were performed in three biological replicates with three technical replicates for each sample. No sample was excluded from the statistical analysis. The final results presented are the outcome of calculating the averages of the GLuc/SEAP ratios in three biological replicates for each reporter plasmid. The luciferase experiment results of 3T3-L1 cells are presented in Supplementary Files S1 and S2 and show the result of three technical replicates within one biological replicate. Reporter plasmids containing fragments E to H were tested in 3T3-L1 cells to confirm the results obtained in HEK 293A cells. Data are shown in Supplementary File S2.

### 4.11. RNA Extraction and cDNA Synthesis

Flash-frozen whole brains of 6-week-old BFMI, B6N, AKR, and SJL mice that had been stored at −80 °C were used for RNA extraction. The brain was disrupted by grinding with a pestle and mortar to a fine powder in the presence of liquid nitrogen. Approximately 30 mg of the ground tissues were used for total RNA isolation using the NucleoSpin RNA isolation (MACHEREY-NAGEL, Düren, Germany) as described in Hesse et al. [53]. The LunaScript^®^ RT SuperMix Kit (New England Biolabs, Ipswich, MA, USA) was used for cDNA synthesis following the manufacturer’s protocol using 1 µg of total RNA as input for each reaction.

### 4.12. Real-Time PCR

Real-time PCR (RT-PCR) was carried out to compare the expression of *Bbs7* in the brain of 6-week-old BFMI (four samples), B6N (four samples), AKR (five samples), and SJL mice (five samples). Takyon™ No ROX SYBR 2X MasterMix blue dTTP (Eurogentec, Seraing, Belgium) was used for RT-PCR. For each sample 2.5 µL Takyon™ MasterMix was used followed by the addition of 0.8 µL primer pair (10 µM, *Bbs7* and *ACTB* primers in Supplementary File S1, Table S1), 1 µL of cDNA, and 1.1 µL nuclease-free water resulting in a 5 µL reaction volume. The PCR cycles comprised: 95 °C for 3 min, followed by 40 cycles consisting of 95 °C for 10 s, 60 °C for 20 s, and 72 °C for 40 s. The RT-PCR was performed in three biological replicates with three technical replicates for each sample. The average CT from the three technical replicates within each biological replicate was calculated. Afterwards, the average CT of the three biological replicates was calculated for each sample (B6N, BFMI, AKR, SJL) and then the relative expression level was calculated using the 2^−ΔΔCT^ method [54]. The fold change of BFMI/B6N was calculated to show the differences in the quantity of *Bbs7* expression between BFMI and B6N (Supplementary Files S4 and S5).

## 5. Conclusions

The results of this study based on the dual-luciferase assays imply that the BFMI allele of SNP rs29947545 is the causal variant for the reduction in the expression of *Bbs7* in transfected cells. However, two-thirds of the mouse strains in the Mouse Genome Project share the BFMI SNP allele in their 5′ UTR of the *Bbs7* promoter region, but are not obese. Hence, we conclude that the other mouse strains carry an additional DNA sequence variant that compensates for the SNP effect and that is missing in BFMI mice and the cell lines tested. This indicates that in mice where the compensatory mechanism does not exist, the BFMI promoter variant could be causal for the down-regulation of *Bbs7* and causing obesity.

This study confirms the relevance of *Bbs7* for obesity. Furthermore, we conclude from the significant effect of a SNP in the promotor of *Bbs7* on gene expression, that sequence variants in the regulatory regions of *Bbs7* in humans should be studied more intensively in order to better understand the occurrence of obesity in association with the Bardet–Biedl Syndrome. Studying regulatory regions can help us better understand complex traits such as obesity. Our results provide fundamental information on the transcriptional regulation of *Bbs7* for future studies to fully unravel the molecular mechanism leading to obesity.

## Figures and Tables

**Table 1:** Sequence variants in the selected 949 bp promoter region including the 159 bp 5′ UTR in BFMI, B6N, AKR, and SJL. The causal SNP rs29947545 responsible for the reduction in expression in the dual-luciferase assay is shown in bold.

Sequence Variant Number	rs ID	BFMI	B6N	AKR	SJL
1	rs232831355	**G**	**A**	**G**	**G**
2	rs247169908	G	T	G	G
3	rs578752359	T	C	C	C
4	rs47275624	C	A	C	C
5	rs47931207	G	A	G	G
6	rs46590687	T	G	T	T
7	rs47190609	T	G	T	T
8	rs48376078	T	G	T	T
9	rs51982785	A	G	A	A
10	rs225736719	A	G	A	A
11	rs247385341	GCGAAGCTCCA	GCGAAGCTCCAGCGAAGCTCCA	GCGAAGCTCCAGCGAAGCTCCA	GCGAAGCTCCAGCGAAGCTCCA
12	rs227723138	G	T	G	G
13	rs51089963	C	A	C	C
14	rs29947551	A	G	A	A
15	rs29947548	C	G	C	C
**16**	**rs29947545**	**C**	**T**	**C**	**C**

## Data Availability

The datasets supporting the conclusions of this article are included within the article and its Supplementary Files. DNA sequencing data were deposited at the NCBI Sequence Read Archive (SRA) under BioProject ID: PRJNA717237 and are available at https://www.ncbi.nlm.nih.gov/bioproject/717237 (accessed on 25 March 2021).

## Supplementary Materials

The following supporting information can be downloaded at: https://www.mdpi.com/article/10.3390/ijms232113018/s1.
