# Supplementary material for: A 5′ UTR Mutation Contributes to Down-Regulation of Bbs7 in the Berlin Fat Mouse
Source: Int J Mol Sci. 2022 Oct 27;23(21):13018. doi: 10.3390/ijms232113018 (PMC9658298; doi:10.3390/ijms232113018)
Supplement: Supplementary file 1 [file ijms-23-13018-s001.zip › supplementary file 1.pdf]

Supplementary data

A 5’ UTR mutation contributes to down-regulation of *Bbs7* in the Berlin Fat Mouse

Kourosh Mohebian[1], *Deike Hesse*[1], *Danny Arends*[1], *Gudrun A. Brockmann*[1] †,

[1] Albrecht Daniel Thaer-Institut für Agrar- und Gartenbauwissenschaften, Humboldt-Universität zu Berlin, Unter den Linden 6, 10099 Berlin, Germany

† Corresponding author

Address for correspondence:

Gudrun A. Brockmann

Animal Breeding Biology and Molecular Genetics

Albrecht Daniel Thaer-Institut

Humboldt-Universität zu Berlin

Unter den Linden 6

10099 Berlin

Germany

Phone: 0049 30 2093 49872

Fax: 0049 30 2093 6397

E-mail: gudrun.brockmann@hu-berlin.de

Primer	Sequence	melting temperature
Prb 1	5’ TTAGCTCACTGTGAAGCCCC 3’	60°C
Prb 2	5’ ACCAGGAGTAGAACAAGGCA 3’	58°C
Prb 3	5’ AGATAGAACCAGATCTTGGaattCACCAGGAGTAGAACAAGGCA 3’	67°C
Prb 4	5’ AGCTCGGTACCAagctTTTAGCTCACTGTGAAGCCCC 3’	69°C
Prb 17	5’ AGATAGAACCAGATCTTGgaattcCTGTCCTGGAACTCACTTTG 3’	67°C
Prb 21	5’ AGCTCGGTACCAagctTAAGCCTTCCCTCGGAGA 3’	57°C
Prb 22	5’ AGATAGAACCAGATCTTGGaattCGCGAAGCTCCAAGCAG 3’	56°C
Bbs7-fwd	5’ GCTACTTATCGGTGCCAGGCAA 3’	62°C
Bbs7-rev	5’ GTGGTATTGGCGAACCTGACAG 3’	60°C
Actb-fwd	5’ CTAAGGCCAACCGTGAAAAGAT 3’	60°C
Actb-rev	5’ CACAGCCTGGATGGCTACGT 3’	62°C

Table S1: List of primers used in this study. Sequences in lowercase letters represent relevant restriction enzyme sites.

Primer combination	Application
Prb 1+Prb 2	Amplifying promoter region of BFMI/B6N for plasmid **D**
Prb 3+Prb 4	Addition of overhangs to BFMI/B6N sequence for plasmid **D**
Prb 17+Prb 21	Amplifying promoter region+ overhangs of BFMI/B6N for plasmid **A**
Prb 21+Prb 22	Amplifying promoter region+ overhangs of BFMI/B6N for plasmid **C**
Prb 21+Prb 3	Amplifying promoter region+ overhangs of BFMI/B6N for plasmid **B**
Bbs7-fwd	Real time PCR
Bbs7-rev	Real time PCR
Actb-fwd	Real time PCR
Actb-rev	Real time PCR

Table S2: List of primers used in this study and their purpose of use.

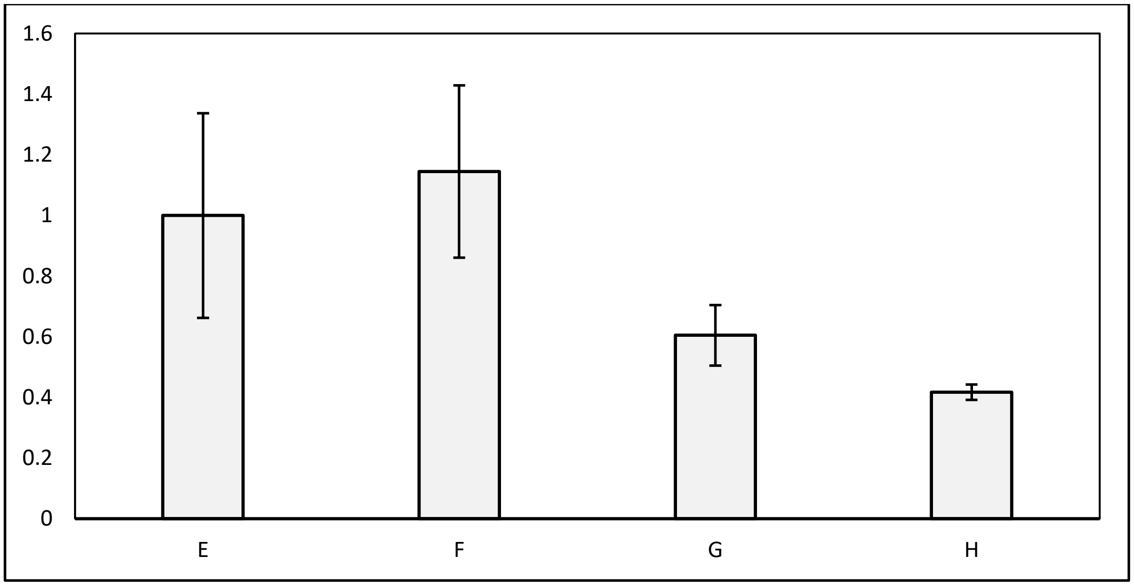

Figure S1: bar chart shows the normalized luciferase expression (GLuc / SEAP ratios) of the tested promoter fragments (E to H) in 3T3-L1 cells. We observe similar results to transfected 293 A HEK cells which is significantly reduced expression in G and H fragments ($FC_{(G/E)}$ = 0.60, $FC_{(H/E)}$ = 0.41), but that is not the case for fragment F ($FC_{(F/E)}$ = 1.14). Normalized luciferase expression (GLuc / SEAP ratio) was plotted as mean ± standard deviation of triplicate samples.
